# Enhancing the MEP Coordination Process with BIM Technology and Management Strategies

**DOI:** 10.3390/s22134936

**Published:** 2022-06-30

**Authors:** Ya Hui Teo, Jun Hong Yap, Hui An, Simon Ching Man Yu, Limao Zhang, Jie Chang, Kang Hao Cheong

**Affiliations:** 1 Science, Mathematics and Technology Cluster, Singapore University of Technology and Design, 8 Somapah Road, Singapore 487372, Singapore; yahui_teo@mymail.sutd.edu.sg (Y.H.T.); junhong_yap@sutd.edu.sg (J.H.Y.); 2 Engineering Cluster, Singapore Institute of Technology, 10 Dover Drive, Singapore 138683, Singapore; hui.an@singaporetech.edu.sg; 3 Interdisciplinary Division of Aeronautical and Aviation Engineering, The Hong Kong Polytechnic University, Kowloon 999077, Hong Kong; simon.c.yu@polyu.edu.hk; 4 School of Civil and Hydraulic Engineering, Huazhong University of Science and Technology, 1037 Luoyu Road, Wuhan 430074, China; zlm@hust.edu.cn; 5 College of Life Sciences, Zhejiang University, Hangzhou 310058, China; jchang@zju.edu.cn

**Keywords:** Building Information Modeling (BIM), mechanical, electrical, and plumbing (MEP) services, management strategies, design modeling, construction industry, machine learning, productivity

## Abstract

Building Information Modeling (BIM) has been increasingly used in coordinating the different mechanical, electrical, and plumbing (MEP) services in the construction industries. As the construction industries are slowly adapting to BIM, the use of 2D software may become obsolete in the future as MEP services are technically more complicated to coordinate, due to respective services’ codes of practice to follow and limit ceiling height. The 3D MEP designs are easy to visualize before installing the respective MEP services on the construction site to prevent delay in the construction process. The aid of current advanced technology has brought BIM to the next level to reduce manual work through automation. Combining both innovative technology and suitable management methods not only improves the workflow in design coordination, but also decreases conflict on the construction site and lowers labor costs. Therefore, this paper tries to explore possible advance technology in BIM and management strategies that could help MEP services to increase productivity, accuracy, and efficiency with a lower cost of finalizing the design of the building. This will assist the contractors to complete construction works before the targeted schedule and meet the client’s expectations.

## 1. Introduction

Buildings are constructed with various complicated structures, just like the methodological arrangement of components in humans [1]. A building is composed of structural, envelope, interior, and mechanical, electrical, and plumbing (MEP) systems. Mechanical, electrical, and plumbing (MEP) systems are the most complicated components, as they need to run throughout the whole building [2]. MEP systems provide services to the building and its occupants to address specific work requirements and/or create a congenial indoor environment so that the building occupants can stay in the building for a prolonged period. An efficient, superiorly designed MEP can promote sustainability, reduce environmental damage, and provide a better working environment for the building occupants in terms of health, thermal and visual comfort, and wellbeing [2,3]. Examples of MEP systems are heating, ventilation, and air-conditioning (HVAC), and utilities, plumbing, and sanitary services. The performances of thermal, indoor air quality (IAQ), acoustic, visual, spatial, and building integrity are closely related to MEP systems.

As the respective services have their codes of practice to follow, MEP services are technically more challenging to coordinate in Building Information Modeling (BIM), as compared to the other services such as architectural and structural services [4,5]. In addition, MEP services must place their pipes and ducts within the limited ceiling heights based on the project requirements [6,7,8]. Coordinating all the services is a time-consuming process that requires more than 50% of the project duration [9,10,11,12,13]. This results in having a higher cost of MEP systems due to reworking [14] and cost overruns [15] as compared to the overall cost of the entire building [8,12,16].

BIM is seen as a superior technology compared to traditional CAD, providing a higher level of capability to reduce conflicts in construction sites [17,18,19]. More advantages can be gained by using the BIM/VDC model, including an estimated 20% to 30% in labor savings, minimal reinstallation, reducing conflicts on the construction site, and pre-fabrication that could be carried out before the construction stage, as all relevant parties can attend the coordination meeting to have an efficient discussion [8]. Although BIM modeling is more efficient than 2D software, more research in BIM is needed to reduce manual work and improve clash detection through various technologies and machine learning to accelerate MEP coordination advancement and cost savings. There is also a research gap in terms of coordinating large projects with appropriate management strategies when various technologies are merged into BIM, which involves a large number of people.

The objective of this paper is to review possible advanced technology solutions in BIM that can further improve the current BIM MEP coordination process and examine possible factors that can affect the BIM coordination process. We first present the research findings that are related to potential limitations in BIM MEP coordination faced by current construction industry sectors, followed by analyzing the solutions to identify the best practical solution. We then provide recommendations for future research in for the modern construction industry.

## 2. Overview

We first provide a summary of important concepts of how BIM aids the modern construction industry. We reviewed a total of 85 publications ranging from 1981 to May 2022. As part of the literature review, we focus on the different technologies and/or software that can be used to enhance the BIM MEP coordination process, as well as automation in BIM—to speed up the clash detection process by removing irrelevant clashes—and strategies to enhance BIM MEP coordination management techniques.

### 2.1. BIM Development Overview

As there are different services in a building, it is essential to ensure that there are no clashes when the contractors install their respective services in the building. BIM software is widely used in the modern construction industry to coordinate the different services involved, such as architecture, structural, and MEP systems for energy efficiency calculations, project coordination, and operation and maintenance.

**Figure 1:**
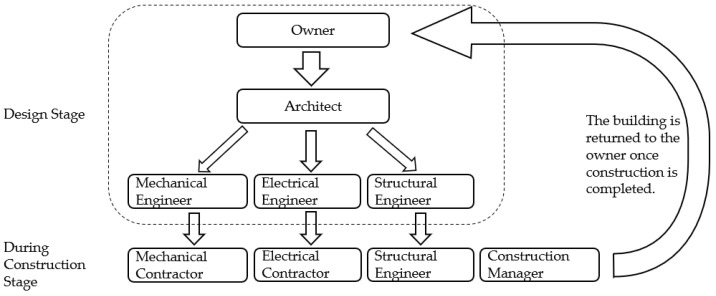
A simple illustration of the general process of the different personnel working in different stages of construction. Overview concept adapted from [20].

Figure 1 depicts a simplified process that demonstrates how various services in the building work. First, the owner presents the concept to the architects. The architectural design is then transmitted down to the respective engineers in the various trades, allowing them to design their services in BIM while also computing the total expenses. Once the architects and consultants have approved the design, the contractors will begin installing their various services in the building. The building is then returned to the owner once construction is completed and approved by the relevant authority.

**Figure 2:**
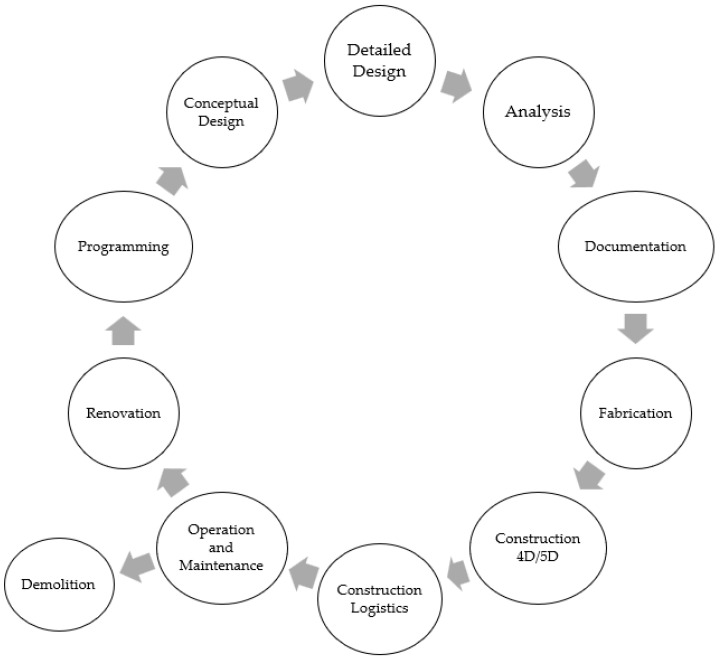
Life cycle of BIM software to showcase the process of generating and managing building data. Image adapted from [25].

**Figure 3:**
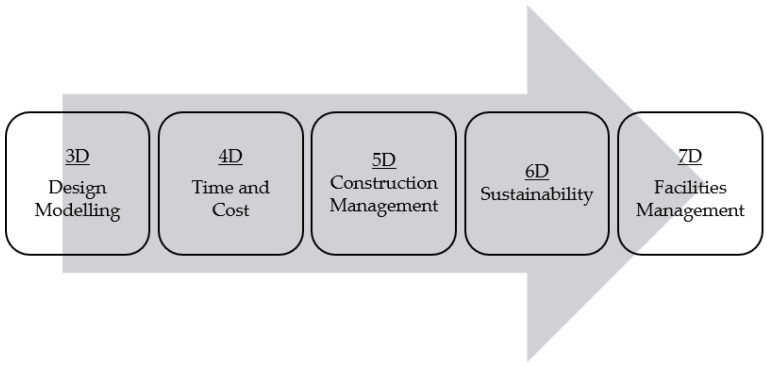
Overview on the different dimensions of BIM where it can be used. Figure modified from [21,23].

BIM is a technique that allows architects and civil, mechanical, and electrical engineers and/or drafters to design, collaborate, consolidate, and show all details on the building/project [21]. BIM can be used in any part of the cycle, from renovation, planning, designing, documentation, and construction, to operation and maintenance as shown in Figure 2 [22]. The different dimensions of BIM, as illustrated in Figure 3, can help to improve the efficiency and productivity in all stages of construction process [21,23]. Further details on the different dimensions of BIM, specifically 3D, 4D, 5D, 6D, and 7D, are explained as follows.

1. BIM 3D (Design Modeling)

This involves all services, such as architecture, structure, and MEP services collaborating on one platform. BIM 3D provides visualization to check for clashes and better communication between all services.

2. BIM 4D (Time)

BIM 4D helps to plan and schedule data on the amount of time needed to complete the project and provide additional information on time variable schedules, such as contracts, project duration, procurement, and cost of contract. This will provide more details on the sequence of the construction and installation process to make site planning manageable. As the information is shared with everyone who is involved in the project, the contractors will be more prepared when it is their turn according to the schedule. This will improve safety and productivity and minimize buffer and lead time on-site if everything goes according to schedule [24].

3. BIM 5D (Construction Management)

BIM 5D can provide a prediction on budget evaluation and cost at the beginning of the project. The cost will also be automatically amended when there are changes to the project. This allows the clients and/or owners to evaluate and track the actual cost used for the project.

4. BIM 6D (Sustainability)

BIM 6D provides an estimate of energy consumption analysis of the building at the initial design stage. The client and/or owner will know the total cost of their building, which includes the construction cost and estimated cost of energy consumption after the building has started operating. They will then have an idea on how to achieve sustainability to further minimize costs on energy consumption. Therefore, BIM 6D can assist in improving sustainability in terms of minimizing energy usage and enhancing operational management when the project is completed.

5. BIM 7D (Facilities Management)

BIM 7D helps building owners track vital asset data, such as information on warranty and technical specifications for future usage in facility operations and management. The building owners will be updated on which areas of the building require maintenance. The relevant information in the BIM 7D model can assist the contractors to improve the maintenance process.

With these progressions, it will be easier to collaborate with other services, e.g., architectural, structural, and MEP services, and integrate the design together to identify and solve clashes before the start of construction stage [26] to avoid conflict on the construction site. Therefore, it is considered as an efficient method to visualize and have an overview of how the design looks.

### 2.2. Constraints in BIM

The use of BIM has increased by 75% between 2009 and 2014. In 2014, nearly three-quarters of the North American building industry used BIM because it allows relevant personnel who are working on the same project to exchange information within a short period of time, improving project quality and completing construction of a building on time [27]. However, there are some limitations in BIM MEP coordination that result in the delay of the construction process due to unfinalized MEP routings as shown in various papers [4,8,12,16,22,28,29]. For instance, Tommelein et al. [22] pointed out that clashes with other MEP services are a norm in the construction industry. One of the root causes is design complexity, which may be ignored if the drafters focus on finishing the drawings and/or BIM models when there is a clash with other services. To resolve the clashes, there is usually a coordination meeting between the drafters and BIM coordinators. As the detected clashes must be updated and resolved in every coordination meeting, it can become time consuming to search and improve on each individual MEP’s location and routing. The other root cause is failure to follow codes of practice. This results when all the services, including architectural and structural, change the design together at the same time. A small change in details and/or design will lead to a major change in MEP [30,31], inconveniencing the MEP sectors. This is because the MEP pipes and ducts are interconnected from the bottom to top of the building. This will result in a waste of coordination effort from previous meetings, and the drafters and/or person-in-charge will have to spend more time redrawing and redesigning the pipes/ducts. The coordination process will then be re-coordinated, resulting in less time for construction.

The virtual representation in BIM could be different from how components, such as pipe connectors, are installed on site. This could be due to the pipes/ducts installed on site being different from those shown in the BIM model in terms of dimensions, shapes, and sizes [24,32]. This could lead to conflicts and installation clashes on site. Thus, it is important to communicate with the drafters and/or person-in-charge to know and understand how the site and standard of practice works to prevent delays in the design process and coordination.

The job scope of BIM coordinators includes checking for clashes in the models and bringing them up to the drafters during meetings [33,34]. The working experience of BIM coordinators will greatly affect the progress of MEP coordination [35]. For instance, novice BIM coordinators lack experiential knowledge [36]; these coordinators may neglect designing according to the code of practice while the drafters focus on achieving zero clashes. The Registered Inspector (RI) will then visit the site when the building is completed and ensure that everything follows the code of practice. However, the contractors will have to dismantle and re-install at appropriate places if it does not meet the conditions. This will further delay the construction process and increase cost expenses of the contractors and owners. As a result, it will be beneficial to incorporate advanced technology into BIM to verify if the designs adhere to the guidelines in order to reduce manual works.

Other than the coordination process, the cost of full-functionality BIM software can be costly for a small designing company. It is more economical to outsource the drawings to a company that has skillful drafters and software to save costs, rather than buying the software and hiring drafters.

### 2.3. Technologies/Software to Enhance BIM MEP Coordination

-
**Revit’s built-in functions**

It is known that at least 60% of organizations execute their coordination operations using a BIM-based strategy [37]. New technologies are frequently employed by building professionals to improve the MEP coordination process in BIM [38]. The new BIM technology has also improved, for example, leveraging on Application Programming Interfaces (APIs) and visual programming to reduce manual work. The Revit API can also be utilized to improve and automate the coordinating process through two stages [39].

-
**First stage**

Focuses on automated ceiling space that is specially designed to aid MEP engineers in the analysis of available and congested ceiling space for MEP components.

-
**Second stage**

MEP engineers develop rule-based routing with the Autoroute Add-In function to automate and enhance the productivity of the MEP coordination process. The Autoroute Add-In function will send an error to the designer immediately if pipes/ducts that flout the standard code of practice are being drawn. 

Clearly, the Revit API can aid in the design process becoming more systematic and less laborious, with fewer reported clashes and coordination meetings. Next, BIM MEP models can be built automatically in using transfer laser scanning data through Dynamo, a Revit visual programming plug-in, to auto-detect and auto-recognize the building’s installations [40]. This technology is especially useful in as-built drawings, where it can be substituted for manual works in revising and updating the plans from the building’s original design to the completion of the construction process, which is a tedious and time-consuming procedure. This methodology has been used in three real case studies in Hong Kong, and it has achieved 91.3% accuracy in reconstructing BIM models [40]. This can also assist the drafters in improving the virtual representation and accuracy in the BIM model. However, this only benefits as-built projects and facility management, whereas the MEP services are installed on site.

-
**A combination of BIM technology and cloud computing interface**

BIM MEP coordination is an essential step in the design stage, as BIM could detect clashes before the contractors install their respective services on the construction site [5,6,41,42]. For instance, Lee et al. [43] adopted both BIM technology and cloud computing interfaces to combine the drawings from all services during the design stage. The benefits of a cloud computing interface include incorporating traditional 2D drawings, managing 3D BIM models and parameters, collaboration with BIM at a lower cost, and improvement of computational capability [44,45]. BIM provides clash detection and clarification reports when all services are linked into a single 3D model. Therefore, BIM technology has greatly helped the authors in improving the productivity and construction quality, allowing the case study to be completed on time.

-
**Clash detection technologies**

One of the current practices in BIM coordination is using Naviswork software to integrate models created by all services and detect clashes based on the user’s settings [33,46]. Clash avoidance, enhanced clash detection, and clash filtering are some of the approaches for minimizing irrelevant clashes [46]. Chahrour et al. [47] state that clashes occur due to model inaccuracy, design mistakes, and uncertainty, leading to delays in the construction progress. A holistic approach to reduce the number of irrelevant clashes is presented in Table 1. Despite improved clash algorithms [48,49,50,51,52,53] and machine learning [54], there is still the possibility that the algorithm will detect false positives, such as creating a list of duplicated clashes in the same area [16,55]. Therefore, Hu et al. [46] suggested speeding up the query process by using a component dependent network (CDN) and Industry Foundation Classes (IFC) to query geometric information, as well as employing bounding volume hierarchy (BVH) to avoid unnecessary comparisons. CDN methodologies consist of determining critical spatial relations such as topology, orientation, and distance for clash management, followed by creating algorithms to investigate dependent relations from BIM models, and lastly, generating a component dependency network and storing it to a database. The authors tested their methodology on a real case study, and it proved that irrelevant clashes had reduced by 17%. With the aid of a CDN to group relevant clashes automatically, the user was allowed to view the clash detection in a glance, as the reported clashes had decreased more than 50%. Therefore, the coordination time would be shortened, and would speed up the construction process.

### 2.4. Review on Strategies to Enhance BIM MEP Coordination Management Techniques

Other than improving technologies, project success also depends on the coordinating methodologies used [30,31], type of project, and the practice in different countries. Back in 2011, about 73% of the construction industry in China does not use BIM to coordinate related services [57]. BIM is only used at certain stages, such as the preliminary, detail design, and construction stages for large-scale projects [58]. Chinese enterprises may be hesitant to modify existing practices, as the cost of BIM software is relatively high compared to other relevant costs, and there is also a lack of encouragement for them to learn BIM skills [59,60]. According to a survey conducted by Jin et al. [61], the issues faced in both Shenzhen and the United Kingdom (UK) include a lack of BIM experience or abilities, which may result in miscommunications between the receivers and senders due to improper model interpretations [62]. This implies that there is a need for personnel working in the construction trade to receive BIM training from industry professionals. With the aid of government encouragement and mandates, an estimated 67% of Chinese firms is beginning to implement BIM as of 2014 [61]. Yung et al. [63] describe that there are also different workflow practices in China and United States (US). China’s methodology involves architects and structural and MEP engineers in the same organization. Thus, the design process will be easier and faster to complete for construction to proceed. On the other hand, the US has a different approach, where the clients will usually outsource to either consultants or subcontractors. They will shoulder additional accountability, with the degree of obligation varying based on the type of project, such as build-only or design-and-build. Subcontractors are now held to a higher level of responsibility in the construction sector than others, for example, the consultants.

Lee et al. [64] did a comparison between a parallel and sequential design coordination approach for their pharmaceutical building case study located in the USA. In the parallel design coordination approach, all services, including MEP services, will update and coordinate their services in the 3D model at the same time. MEP coordination tasks in the sequential approach, as opposed to the parallel approach, are prioritized and carried out using the Sequential Comparison Overlay Process (SCOP) [7]. The results proved that the sequential design coordination approach is a better strategy in BIM coordination, as it has the least number of clashes and coordination meetings. As there was less workload for the coordinator, the productivity of the sequential design coordination approach, measured in speed, was about three times faster than the parallel design coordination approach. 

Apart from the BIM coordination process, Tillmann [65] suggested that social networking, such as the Last Planner System (LPS), is essential in aiding team building by providing a framework for better communication and responsibility, as well as assisting the project team in concentrating on problem solving. This could minimize the percentage of rework on the construction site and enhance work productivity.

**Figure 4:**
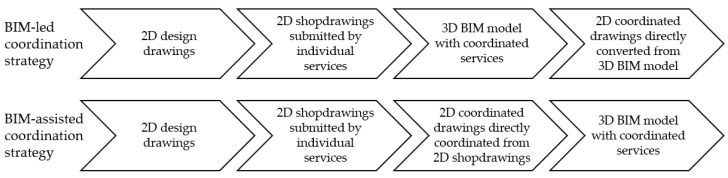
A comparison between BIM-led and BIM-assisted coordination strategies, which use a mixture of 2D drawings and a 3D BIM model modified from [30].

BIM can help all services in the construction industry speed up the process of MEP coordination and replace the process of drawing in 2D software. For instance, Park et al. [30] made a comparison between two different types of workflows on a project of two buildings in Sweden that uses a mixture of 2D software and BIM, as illustrated in Figure 4. One of the projects, using 2D software to make shop drawings for reviews and submissions, used a BIM-assisted coordination strategy. The subcontractors commenced work once the MEP engineers approved the shop drawings. The coordinated drawings were then reviewed in the BIM model as an assistance for checking for clashes. Not only did it take longer to redo and change shop drawings after issues were discovered in the BIM model, but there were also disagreements on the construction site, and pipes and ducts had to be dismantled. The other building utilized a BIM-led coordination strategy to focus on coordinating all trades in the BIM models. 2D drawings were only generated for submission purposes when the coordination was complete and error-free. Based on the comparison of 2 strategies, the BIM-led coordination strategy proved to be better with a shorter coordination time, less design change, and higher quality of design. In addition, 2D drawings may become outdated in the future, and with the aid of current advanced technology, it may be a good practice to use BIM to lead the project since it can improve the productivity and efficiency of the construction industry.

## 3. Discussion

The presented methodologies of several case studies in Section 2.3 and Section 2.4 could solve the constraints and improve accuracy and efficiency in the process of workflows in BIM design coordination. Management techniques play a significant role in the BIM design coordination process, which is at least 28% faster than current practices [30]. With these methodologies and management strategies, we now provide suggestions that may help in overcoming the restrictions described in Section 2.2, as well as in meeting our goals of boosting productivity, accuracy, and efficiency while reducing costs in finalizing the design of the building, as shown in Table 2 below.

**Figure 5:**
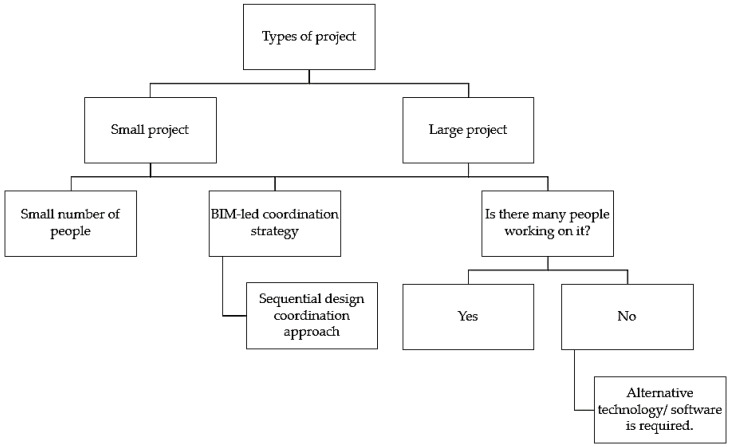
A summary of management strategies recommended for small and large projects. The similarity between small and large projects is that both are suitable for using a BIM-led coordination strategy [30] and sequential design coordination approach [64]. Alternative technology/software in BIM is required when there is a limited number of people working on a large project.

The construction process will be faster when there are fewer parties engaged in a project, when MEP coordination tasks are prioritized and completed in a sequential manner, and when 3D BIM models are created to generate 2D coordinated drawings for submission. In this way, the BIM design coordination management will be more organized, and the respective services will have a better understanding of the project’s requirements. This would lead to a smoother and faster flow of constructing and installing the respective components in the construction site, with minimized conflicts and clashes on site. However, it is not possible to have a small number of people working on a big commercial project. Therefore, technologies and/or other software are required to speed up the BIM design coordination process when there is limited manpower working on the project. A summary of the management strategies that are suitable for both small and large projects is shown in Figure 5 below.

There are numerous papers that explore ways to speed up the design process of architecture and/or structure of the building [66,67,68,69]. For instance, Wang et al. [70] proposed integrating lean construction with BIM, which includes an automatic precast element design engine (APEDE), to improve precast concrete productivity and design. Their findings reveal an increase in efficiency of more than 68 percent and a reduction in physical labor of about 6 h. Next, Liu and Shi [71] use a similar technique by using lean construction with BIM to undertake quality control with KanBIM technology, to increase construction quality while lowering costs. With a combination of both studies, this could be a worthwhile research opportunity to investigate the technique for improving MEP’s design process, engagement, and collaboration with other trades, since it promotes sustainability and reduces reworks for the relevant parties in the construction industry. On the other hand, Lim et al. [72] presented a real case study using Grasshopper, a visual programming plug-in of Rhinos software, to generate a 3D BIM model from ArchiCAD 2D drawings automatically. This mainly helps to speed up the process of architect drawings through GhPythonm, an add-on from Grasshopper, for programming purposes. However, there are some limitations in the automation process, such as 2D drawings needing to be cleaned with correct layer names before they are processed. The 3D model requires manual editing when it cannot be detected during the automated process.

With the aid of advanced technology, automation in the BIM coordination process could reduce manual work in modeling, as BIM design coordination is the most complicated process, which can lead to time and cost overruns and construction delays. Lin and Huang [55] conducted interviews with the senior project managers in Taiwan, and they stated that BIM managers either selectively or completely disregard the assessment of clash reports because it is a time-intensive process and there are high costs in evaluating the clashes. This shows that there is a need to research automation processes and/or other technologies [45,73] to reduce workload in construction industries. For instance, Hu et al. [54] made a comparison between six different types of supervised machine learning algorithms. Jrip-based rule methods performed the best in filtering out irrelevant clashes by using past data.

There are various studies that use machine learning with a hybrid approach in civil engineering [74,75,76,77] to increase prediction accuracy, such as naïve Bayesian classifiers [78]. For instance, Noori Hoshyar [79] presented a machine learning methodology in using the Support Vector Machine (SVM) to classify and predict data [80] on monitoring structures for various types of damages that occur during loading. The algorithms, 2D fast Fourier transform (FFT) and hybrid fuzzy c-mean procedures, were more effective than standard techniques in recognizing and visualizing the cracking in the structure. Although Lin and Huang [55] managed to further explore a hybrid method in combining rule-based reasoning and supervised machine learning in BIM design coordination to detect irrelevant clashes automatically, it is only applicable to structural and pipe clashes. The hybrid method achieves an average prediction performance of 0.96, which is up to a 6% to 17% improvement over the typical machine learning procedure that uses only individual or ensemble learning classifiers, which is a breakthrough in BIM design coordination. However, the hybrid approach necessitates manual dataset labeling, which is a time-consuming process. It was also observed that a good classification performance in machine learning often necessitates a larger training dataset, which allows for a more complicated model with additional features. These machine learning approaches can be studied further in BIM design coordination to reduce the number of clashes and workload in the relevant trades.

BIM is also a versatile software that may be used in conjunction with a variety of technologies, for example, with the Blockchain technology (BCT) to optimize productivity. BCT maintains a database that keeps information methodically and delivers a copy to all parties involved in the project with legality and safety [81]. The integration of BCT and BIM makes it more efficient during the pre-construction stage by clarifying responsibilities, minimizing information misuse, and improving information flow security [82]. However, it is noted that the BCT has several constraints, such as digital contracts with inaccurate information [83] and network performance limitations when large amounts of data are being handled [82]. With the advent of technology, the combination of BCT with BIM is probably a viable solution in the MEP design process that secures a project’s private information while also facilitating workflow in the construction sector [82,84].

**Figure 6:**
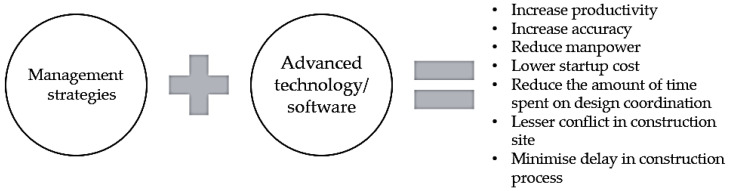
BIM design coordination process in the construction industry will benefit significantly from a suitable combination of management methods and advanced technology.

When sophisticated technologies are introduced into BIM, it is beneficial to make recommendations on coordination techniques to further improve the productivity process in the building sector, as illustrated in Figure 6. For instance, the approaches described in Table 1 are likely to be adequate for using a BIM-led coordination strategy to eliminate at least 50% of irrelevant clashes [46] and time spent on coordination. This allows the engineers to have more time to discuss the issues found with the respective trades and avoid conflict on site.

Furthermore, BIM usage can be extended to facility management (FM). For instance, Cheng et al. [85] presented a more effective maintenance strategy based on a data-driven predictive maintenance schedule that incorporates BIM and the Internet of Things (IoT), with an information layer and an application layer. The information layer collects and combines information from the BIM models, FM systems, and IoT networks, while the application layer comprises four modules (condition monitoring, fault alarming, condition evaluation, and prediction and maintenance planning) for predictive maintenance. When the data are regularly updated, the authors also use machine learning methods such as ANN and SVM in the application layer to estimate the future status of MEP equipment for maintenance planning.

Although BIM is a powerful and well-established international tool for those working in the construction-related industry, the physical site condition may differ from the BIM model due to differences in concrete quality and fabrication of ducts and pipes. Therefore, Zhang et al. [24] stated that the MEP contractors deploy 3D laser scanners to collect and generate structural components’ as-built point cloud model. The data were then synced with 3D BIM models to provide correct MEP manufacturing drawings. A robotic total station was also used in conjunction with 3D BIM models to enable fast and precise MEP system planning for swift installation.

## 4. Conclusions

In conclusion, we have reviewed relevant technology advances and management strategies related to BIM design coordination that can help MEP services to increase productivity, accuracy, and efficiency in finalizing the design of building and construction work. Relying solely on advanced technology is insufficient if management tactics in the BIM design coordination process are poor. We have also summarized possible solutions and the different practices found in other countries that can help to save time and minimize problems that delay the construction process. With the aid of advanced technology, BIM has further improved with visual programming that provides future research directions for a machine learning hybrid method to be incorporated into the BIM design coordination process, to further explore the possibilities of enhancing the workflow and coordination of MEP services on BIM. BIM modeling is projected to be 10% faster and 80% more accurate with the help of Autodesk Revit software, as compared to 2D software [21]. However, more research is required on finding plausible methods to automate dataset labeling, as well as exploring the methodologies that can speed up the work for architectural and structural tools such as Grasshopper and Dynamo. These methodologies can aid in the process on drawing MEP services, increasing productivity, and speeding up the construction process. Other than focusing on clashes, machine learning could further help incorporate the standard code of practice into BIM design that allows all users from different services to learn from each other. However, the critical issue is that it might take a long period of time for the construction industry to use visual programming to speed up the process, as the respective personnel need to learn coding from scratch. It may become complicated and messy when multiple drafters/persons-in-charge are working on the model at the same time. Nonetheless, machine learning and artificial intelligence algorithms in BIM are untapped resources to be utilized in the construction industry at a lower startup cost, and they reduce manpower.

## Figures and Tables

**Table 1:** A summary of technologies and/or software that could help to minimize irrelevant clashes.

Methodologies to Minimize Irrelevant Clashes [46]	Technologies and/or Software
Avoid clashes	Revit Application Programming Interface (API) [39] Automated clash detection [41] Cloud-based shared workspace [43] Evolution-Sensitivity Architecture [56]
Enhanced clash detection	IFC method [49] Bounding volume hierarchy [50] Spheres are used to estimate polyhedral for time-critical collision detection [51] Sphere-trees method [52] Oriented Bounding Boxes (OBB)-trees method [53]
Clash filtering	Differentiate the clashes into soft, hard, and time clashes [22]

**Table 2:** Recommendations to solve the limitations stated in Section 2.2 based on the categories and/or solutions presented in Section 2.3 and Section 2.4.

No.	Limitations in BIM Described in Section 2.2	Categories and/or Solutions Presented in Section 2.3	Management Strategies Presented in Section 2.4
1.	Design complexity, where it is difficult and time consuming to solve the clashes manually.	Revit’s built-in functions help to automate the coordination process and guarantee that the design adheres to industry standards.	The Sequential Comparison Overlay Process (SCOP) [7] could prioritize MEP activities and carry them out sequentially to reduce clashes and coordination meetings, as well as the coordinator’s workload. BIM-led coordination strategies [30] aid in reducing manual work and conflicts on construction site.
2.	The design is not in accordance with the standard code of practice.		
3.	A lot of manual work is required from BIM coordinators and drafters.		
4.	Virtual representation of components is different from the actual components installed on site.	A component dependent network (CDN) and Industry Foundation Classes (IFC) [46] will improve the accuracy of virtual representations by asking for geometric data.	The Last Planner System [65] will aid in reducing miscommunications between engineers and drafters, allowing the drafters to precisely depict the actual components on site.
5.	It is quite expensive for a small designing company to pay for a fully functional BIM software.	A cloud computing interface could collaborate with BIM at a cheaper cost, along with other capabilities such as combining traditional 2D drawings and managing 3D BIM models [44,45].	Outsource to consultants or subcontractors in the same way that the US does [63].

## Data Availability

Not applicable.
